# ﻿*Agapeteshongheensis* (Ericaceae), a new species from Yunnan, China

**DOI:** 10.3897/phytokeys.251.137015

**Published:** 2025-01-20

**Authors:** Chun-Yu Zou, Bing-Mou Wang, Yu-Song Huang, Yi-Hua Tong

**Affiliations:** 1 Guangxi Key Laboratory of Plant Conservation and Restoration Ecology in Karst Terrain, Guangxi Institute of Botany, Guangxi Zhuang Autonomous Region and Chinese Academy of Sciences, Guilin, 541006, Guangxi, China Guangxi Institute of Botany, Guangxi Zhuang Autonomous Region and Chinese Academy of Sciences Guilin China; 2 Laboratory of Systematic Evolution and Biogeography of Woody Plants, College of Nature Conservation, Beijing Forestry University, Beijing, 100871, China Beijing Forestry University Beijing China; 3 Panyu Central Hospital, Guangzhou 511400, Guangdong, China Panyu Central Hospital Guangzhou China; 4 Laboratory of Plant Resources Conservation and Sustainable Utilization & Key Laboratory of Digital Botanical Garden of Guangdong Province, South China Botanical Garden, Chinese Academy of Sciences, Guangzhou, 510650, China South China Botanical Garden, Chinese Academy of Sciences Guangzhou China; 5 South China National Botanical Garden, Chinese Academy of Sciences, Guangzhou, 510650, China South China National Botanical Garden, Chinese Academy of Sciences Guangzhou China

**Keywords:** Morphology, pollen, taxonomy, Vaccinieae

## Abstract

*Agapeteshongheensis*, a new species of Ericaceae from Yunnan, China, is described and illustrated. This new species resembles *A.mannii* and *A.hosseana*, but differs from the former by its linear or narrowly oblong and bullate leaf blade with a strongly recurved leaf margin and obvious reticulate veinlets adaxially, and larger flowers with yellow green and glabrous corollas and longer stamens, and can be distinguished from the latter by having glabrous twigs, linear or narrowly oblong leaf blades, yellow green corollas and exerted style.

## ﻿Introduction

*Agapetes* D. Don ex G. Don (Ericaceae) is a genus of flowering plants predominantly distributed in the Asian subtropical monsoon region. It comprises ca. 115 species globally, with around 63 species currently circumscribed in China, distributed in south-west China (POWO 2024). In the last 10 years, 12 new species of this genus have been described (Tong and Xia 2014; Tanaka et al. 2016; Tong 2016; Zhou et al. 2017; Tong et al. 2019, 2021, 2022; Yang et al. 2019, 2022, 2023, 2024). The diversity within the genus requires further investigation.

During a field expedition to the subtropical forests in southeastern Yunnan in 2023, an epiphytic *Agapetes* plant with large root tubers caught our attention. After a detailed examination of morphological characters with congeneric similar species, we concluded that this species is new to science, as described and illustrated below.

## ﻿Materials and methods

The measurements and descriptions of the new species were based on both living and dried specimens collected from the wild. Measurements were performed with a ruler and small plant parts were observed and measured under a Scanning Electron Microscope (ZEISS EVO18). To discern morphological distinctions from related species, we referred to pertinent literature on species ranging from East Asia to South-West China, Indochina, and South-East Asia, as well as relevant herbarium specimens (Airy Shaw 1935, 1948a, b, 1958, 1960; Stevens 1985; Banik and Sanjappa 2007; Murata et al. 2023). The type specimens were deposited at IBK and IBSC (Thiers 2019).

## ﻿Taxonomy

### ﻿ Agapetes hongheensis

Taxon classificationPlantaeEricalesEricaceae

Y.H. Tong & C.Y. Zou sp. nov.

45A015BC-3D06-54DF-BA3C-F3CB5C8AD178

urn:lsid:ipni.org:names:77355470-1

Fig. 1

#### Type.

China • Yunnan, Honghe Hani and Yi Autonomous Prefecture, Yuanyang County, Ezha Town, Yanjia Village, epiphytic on trees in the forest, elevation 1872 m, 12 Sep 2023, *C.Y. Zou & J.Q. Huang ZCY5280* (holotype: IBK!; isotype: IBSC!).

#### Diagnosis.

**Figure 1.**
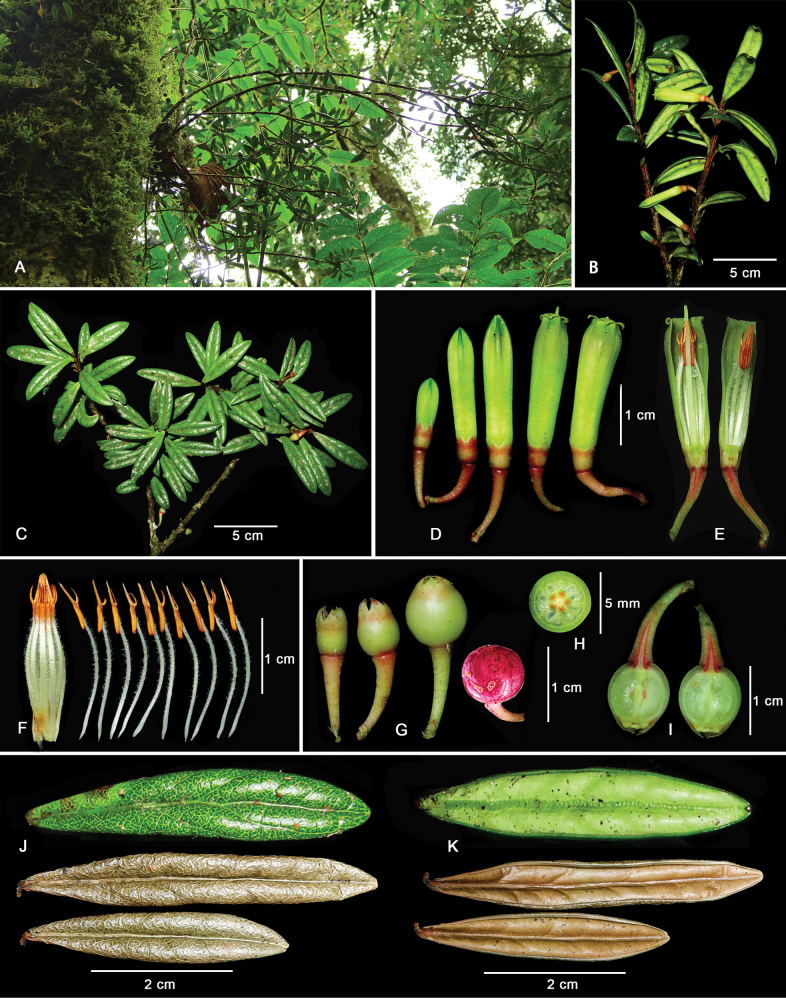
*Agapeteshongheensis***A** habit **B** flowering branch **C** fruiting branches with young fruits **D** flowers of different development periods **E** longitudinal section of flower **F** androecium and lateral view of stamens **G** fruits of different development periods **H** transverse section of young fruit **I** longitudinal section of young fruit **J** adaxial view of fresh and dry leaves **K** abaxial view of fresh and dry leaves. Photographed by Chun-Yu Zou based on the holotype. Scale bars: 5 cm (**B, C**); 2 cm (**J, K**); 1 cm (**D, E, F, G, I**); 5 mm (**H**).

**Figure 2.**
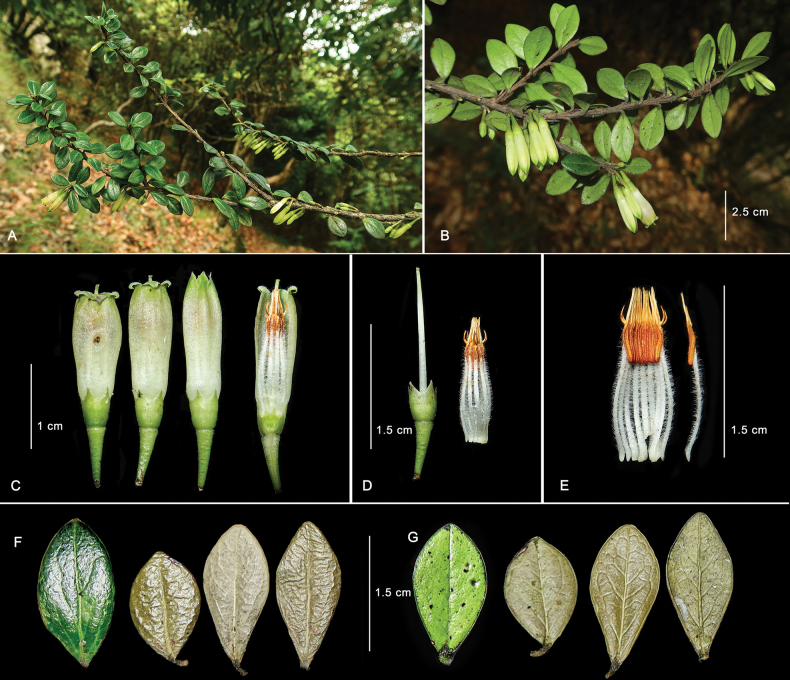
*Agapetesmannii***A, B** flowering branches **C** flowers of different development periods (left) and longitudinal section of flower (right) **D** flower with corolla and stamens removed (left) and androecium (right) **E** adaxial and lateral view of stamens **F** adaxial view of fresh and dry leaves **G** abaxial view of fresh and dry leaves. Photographed by Chun-Yu Zou based on *Chun-Yu Zou and Jin-Quan Huang ZCY5446* (IBK). Scale bars: 2.5 cm (**B**); 1.5 cm (**D, E, F, G**); 1 cm (**C**).

*Agapeteshongheensis* is similar to *A.mannii* Hemsl. (Fig. 2) and *A.hosseana* Diels, but differs from the former by its linear or narrowly oblong and bullate leaf blade with a strongly recurved margin (Fig. 1B, C) and obvious reticulate veinlets adaxially (Fig. 1J, K), and larger flowers with yellow green and glabrous internal surface of the corollas, and can be distinguished from the latter by having glabrous twigs, linear or narrowly oblong leaf blades, yellow green corollas and exerted style.

#### Description.

Shrubs epiphytic, ca. 2 m tall. Root tubers spindle-like or globose, 20–30 cm in diameter. Stems erect. Twigs slightly angled, glabrous. Leaves spirally alternate, often crowded at the apex of branchlets; petiole ca. 1 mm long, glabrous; leaf blade linear or narrowly oblong, 3.0–5.5 × 0.5–1.0 cm, length width ratio 4.95/1, leathery, glabrous, adaxially bullate, midvein raised on both surfaces, lateral veins raised, veinlets conspicuous, abaxially smooth, base cuneate, apex rounded, margin recurved, entire. Flowers solitary or 2, axillary or on old leafless stem; bracteoles 2, basal, triangular, ca. 0.8 mm long, glabrous; pedicles dark red, 7–11 mm long, expanded upwards, glabrous or sparsely pubescent. Calyx tube pastel red, ca. 3 mm long, sparsely pubescent; limb divided nearly 1/3; lobes triangular, ca. 2 × 1.5 mm, apex acute. Corolla yellowish green, tubular; tube 1.9–2.3 cm long, glabrous on both sides; lobes recurved, triangular, ca. 2 mm long. Stamens 10, 1.8–1.9 cm long; filaments flat, 1.2–1.3 cm long, densely pubescent; anthers ca. 6 mm long; thecae ca. 3 mm long; tubules ca. 3 mm long, with 2 erect spurs, spurs ca. 2 mm long. Style glabrous, 1.8–2.3 cm long, exerted from corolla; stigma punctate. Berries globose, almost glabrous, bright red to purple when ripe, 5–8 mm in diameter.

#### Phenology.

*Agapeteshongheensis* is known to flower in September-October and fruit in January the next year.

#### Etymology.

The species epithet denotes where it was found, viz. Honghe Hani and Yi Autonomous Prefecture.

#### Distribution and habitat.

This new species is known only from the type locality, i.e. Yuanyang County, Yunnan Province, China. It grows on the trunks of trees like *Phoebemacrocarpa* C. Y. Wu in evergreen broad-leaf forests at an elevation of 1900 m.

#### Similar species and notes.

*Agapeteshongheensis* is assigned to Agapetesser.LongifilesAiry Shaw (1935: 25), which is characterized by fasciculate inflorescence or solitary flower, and elongated filaments longer than anthers and spurred anther (Airy Shaw 1935, 1959). In the ser. Longifiles, except our new species, only two other species, viz., *A.mannii* and *A.hosseana*, have leaf blade with a round apex and tubular corolla with a glabrous external surface. These two species can be easily distinguished from our new species due to their very distinct morphology of the leaf blade and twigs, and corolla color and length. A more detailed comparison among these morphologically similar species is presented in Table 1.

**Table 1.** A morphological comparison among *Agapeteshongheensis*, *A.mannii* and *A.hosseana*. Data of *A.mannii* and *A.hosseana* are from Fang and Stevens (2005) and Watthana (2012).

Character	*A.hongheensis*	*A.mannii*	*A.hosseana*
Twigs	Slightly angled, glabrous	Terete, densely puberulous	Terete, setose
Leaf blade	Linear or narrowly oblong, 3.0–5.5 × 0.5–1.0 cm	Obovate, elliptic or spathulate, 1.1–2.5 × 0.5–1.1 cm	Oblanceolate, obovate, oblanceolate-oblong to elliptic, 1.7–3.5 × 0.6–2 cm
Veinlets on adaxial surface	Conspicuous	Inconspicuous	Inconspicuous
Pedicle length	Ca. 1 cm	0.4–0.8 cm	1–1.8 cm
Calyx limb	Divided nearly 1/3, lobes triangular	Divided nearly 1/2, lobes subtriangular	Divided 1/3 to 1/2, lobes short triangular
Corolla color	Yellow green	White or greenish white, rarely reddish	Bright red, orange-red, rarely light green
Corolla indumentum	Glabrous on both sides	Glabrous outside, pubescent inside	Glabrous on both sides
Corolla length	1.9–2.3 cm	Ca. 1.6 cm	1.5–2.3 cm
Stamen length	1.8–1.9 cm	Ca. 1.5 cm	1.3–1.7 cm
Filaments	Densely pubescent	Densely pubescent	Sparsely pubescent
Style	Exerted	Exerted	Included

**Figure 3.**
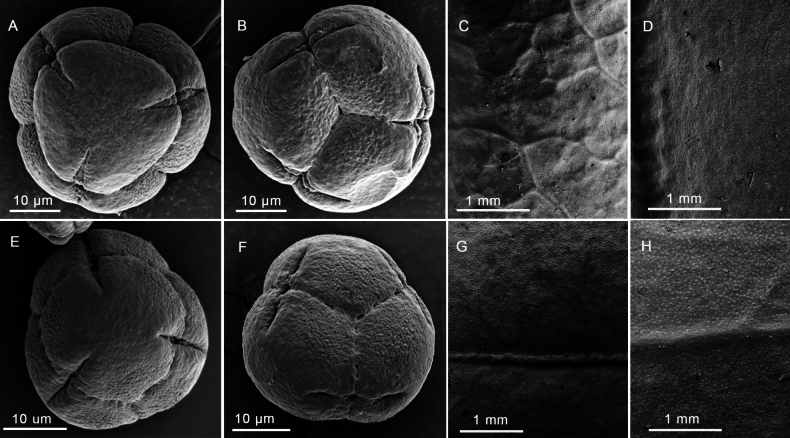
**A, B** the apocolpium ornamentation and the mesocolpium ornamentation of the pollen grains of *Agapeteshongheensis*, from holotype **C, D** the adaxial and abaxial leaf epidermis of *A.hongheensis* under stereoscope with a magnification of 20X **E, F** the apocolpium ornamentation and the mesocolpium ornamentation of the pollen grains of *A.mannii*, from *Chun-Yu Zou and Jin-Quan Huang ZCY5446* (IBK) **G, H** the adaxial and abaxial leaf epidermis of *A.mannii* under stereoscope with a magnification of 20X.

We also tried to perform a microscopic comparison of leaf epidermis and pollen exine sculpture morphology of *A.hongheensis* (Fig. 3A–D) and *A.mannii* (Fig. 3E–H). The midveins of both *Agapeteshongheensis* and *A.mannii* are prominently elevated adaxially and abaxially. The veinlets of *A.hongheensis* are distinctly raised adaxially and form a reticulate pattern (Figs 1J, K, 3C), while the veinlets of *A.mannii* are not obvious (Figs 2F, G, 3G). The pollen grains of *Agapeteshongheensis* are sub-spheroidal tetrahedral tetrads with a diameter of 36.81 ± 1.47 μm, and the ornamentation of both apocolpium and mesocolpium are granulate (Fig. 3A, B). By comparison, the tetrads of *A.mannii* are of a smaller size, averaging 35.53 ± 1.54 μm in diameter, with a similar granulate ornamentation on both apocolpium and mesocolpium (Fig. 3E, F). There seems to be little difference between the two species on pollen exine sculpture.

## Supplementary Material

XML Treatment for Agapetes hongheensis

## ﻿Additional information

### Author ORCIDs

Chun-Yu Zou https://orcid.org/0000-0001-6004-6551

Bing-Mou Wang https://orcid.org/0000-0002-1103-2599

Yu-Song Huang https://orcid.org/0000-0003-2693-8518

Yi-Hua Tong https://orcid.org/0000-0002-5034-005X

### Data availability

All of the data that support the findings of this study are available in the main text.
